# Proton conductivity resulting from different triazole-based ligands in two new bifunctional decavanadates†

**DOI:** 10.1039/c8ra02694g

**Published:** 2018-05-22

**Authors:** Jia-Peng Cao, Feng-Cui Shen, Xi-Ming Luo, Chen-Hui Cui, Ya-Qian Lan, Yan Xu

**Affiliations:** College of Chemical Engineering, State Key Laboratory of Materials-Oriented Chemical Engineering, Nanjing Tech University Nanjing 210009 P. R. China yanxu@njtech.edu.cn; Jiangsu Collaborative Innovation Centre of Biomedical Functional Materials, Jiangsu Key Laboratory of New Power Batteries, School of Chemistry and Materials Science, Nanjing Normal University Nanjing 210023 P. R. China; College of Biological and Chemical Engineering, Anhui Polytechnic University Wuhu 241000 P. R. China

## Abstract

Triazole, similarly to imidazole, makes a prominent contribution to the proton conductivity of porous materials. To investigate the effects of triazole-based ligands in polyoxovanadates (POVs) on proton conduction, we designed and synthesized two decavanadate-based POVs, [Zn_3_(C_2_H_4_N_4_)_6_(H_2_O)_6_](V_10_O_28_)·14H_2_O (1) and [Zn_3_(C_2_H_3_N_3_)_8_(H_2_O)_4_](V_10_O_28_)·8H_2_O (2) constructed from the ligands 3-amino-1,2,4-triazole and 1*H*-1,2,4-triazole, respectively, *via* an aqueous solution evaporation method. Surprisingly, complex 1 obtained a superior proton conductivity of 1.24 × 10^−2^ S cm^−1^ under 60 °C and 98% RH, which is much higher than that of complex 2. Furthermore, due to the contribution of the conjugate properties of the ligands to the third-order nonlinear optical (NLO) properties, we also studied its two-photon responses and achieved satisfactory results.

## Abstract 2

To investigate the effects of triazole-based ligands in polyoxovanadates (POVs) on proton conductivity, we designed and synthesized two decavanadate-based POVs.

## Graphical abstract

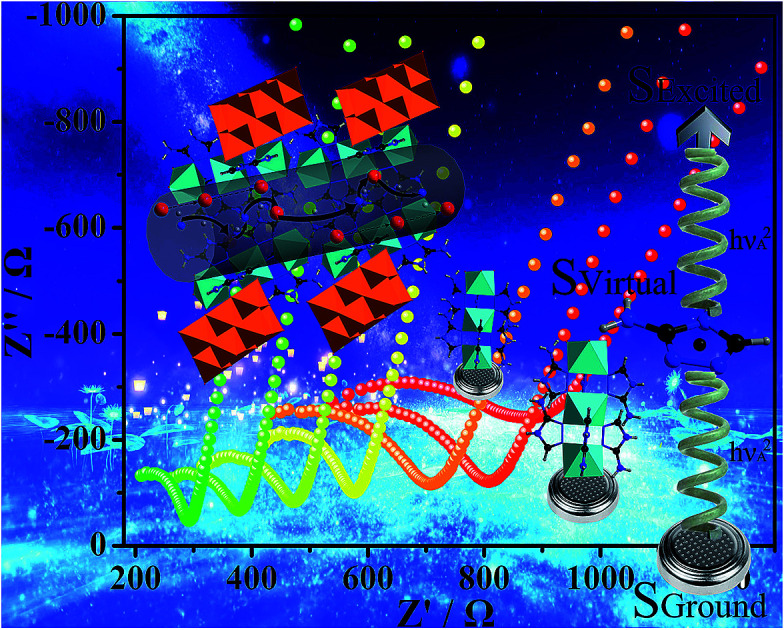

## Introduction

A fuel cell (FC) is a power plant that converts the chemical energy present in fuels and oxidizers directly into electrical energy to provide alternative clean energy.^1^ In particular, proton exchange membrane fuel cells (PEMFCs) as the best option for transportation applications among fuel cells have drawn great interest from quite a few researchers.^2^ Currently, commercial Nafion membranes are the most common due to their superior proton conductivities of 10^−1^–10^−2^ S cm^−1^ at 60–80 °C with 98% relative humidity (RH).^3^ Nevertheless, Nafion membranes cannot retain their high conductivity at enhanced temperatures under comparatively low humidity, and also demand an intricate manufacturing process with high manufacturing costs.^4^ With the development of PEMFCs, there is an urgent need to design and synthesize new porous materials with versatile proton conductivity. Subsequently, due to the prominent chemical stability and low cost of inorganic and composite materials in manufacturing, the proton conductivity performance of these materials has already been exploited to overcome the limitations of polymer membranes.^5^

As a category of significant metal–oxygen clusters composed of transition metals, polyoxometalates (POMs) have aroused the interest of researchers in the field of proton conductivity ascribed to controlled structural design. As we know, in 1979, the first proton conductive material based on a POM was reported by Nakamura, and had high proton conductivities of 0.18 and 0.17 mho cm^−1^ (1 mho cm^−1^ = 10^−2^ S cm^−1^) at 25 °C in the Keggin heteropoly compounds H_3_PM_12_O_40_·29H_2_O (M = Mo, W).^6^ Among the previously reported literature, the highest proton conductivity of 3.64 × 10^−2^ S cm^−1^ was achieved in the Dawson structural POM H_9_P_2_W_15_V_3_O_62_·28H_2_O.^7^ In addition, hybrid POMs with triazole-based ligands have already been investigated, and exhibited predominant chemical stability and favourable proton conductivity properties.^8^ In 2015, Zheng’s group reported the POM-based [H_2_en]_4_[Ni_5_(OH)_3_(L)_3_(en)(H_2_O)(B-α-PW_9_O_34_)]·6H_2_O (en = ethylenediamine) constructed *via* the introduction of a sulfurated triazole-based ligand (L = 1*H*-1,2,4-triazole-3-thiol (H_2_trzS)). This material exhibited decent water stability and possessed a gratifyingly high yield, but had a relatively low proton conductivity value of 2.4 × 10^−4^ S cm^−1^ at 85 °C and 98% RH, which has allowed for a very large development space. Thus, how to design and synthesize new proton-conducting materials with different derivatives of functional species or groups such as triazoles to enhance proton conduction is a crucial objective.^2*b*^

Recently, with the development of bifunctional and multifunctional materials, more and more researchers are concerned with bi- or multi-functionalized POM-based materials, with, for example, electrical, optical, magnetic and catalytic properties.^9^ Among the rest, electro-optical combination in POM-based functional materials with potential applications has received more attention.^9*c*^ As a greatly important part of the optical properties, third-order nonlinear optical (NLO) properties were investigated for the reported POM-based inorganic–organic hybrid complexes,^10^ from which it was undisputed that the conjugated units from organic ligands had a decisive contribution to the third-order NLO responses.

In this context, we designed and synthesized two decavanadate-based POVs on account of the contribution of triazole-based ligands to proton conductivity. Simultaneously, we selected different triazole-based ligands to compare their effects on the proton conductivity of the POVs. [Zn_3_(C_2_H_4_N_4_)_6_(H_2_O)_6_](V_10_O_28_)·14H_2_O (1) and [Zn_3_(C_2_H_3_N_3_)_8_(H_2_O)_4_](V_10_O_28_)·8H_2_O (2) were successfully synthesized with 3-amino-1,2,4-triazole and 1*H*-1,2,4-triazole, respectively, *via* an aqueous solution evaporation method, and were then structurally characterized. A proton conductivity of 1.24 × 10^−2^ S cm^−1^ at 60 °C with 98% RH for complex 1, was obtained using proton conductivity measurements and analyses. Due to the higher number of lattice water molecules moving in a disordered way in 1, the proton conductivity of 1 is much higher than that of complex 2, which could be explained with the Grotthuss mechanism.^2*b*^ More than that, the main effect is that the 3-amino-1,2,4-triazole of 1 has a more protonated –NH_2_ than the 1*H*-1,2,4-triazole of 2, which is ascribed to the vehicle mechanism.^15^ That is to say, the proton conductivities of the two complexes combined two mechanisms, the Grotthuss mechanism and a partial vehicle mechanism. Furthermore, since the N-miscellaneous triazole five-membered ring formed a conjugate unit, an important factor that leads to a third-order NLO response, we also made a thorough inquiry into the third-order NLO properties of the two compounds and obtained satisfactory results.

## Experimental section

### Materials and general methods

A simple and fascinating synthetic method was used in this article. All of the reagents that we used were purchased from Sinopharm and were not further purified. In addition, elemental analyses of C, H, and N were obtained using a Perkin-Elmer 2400 elemental analyzer. KBr pellets with the samples were used to record FT-IR spectra on a Nicolet Impact 410 Fourier transform infrared spectrometer in a range of 4000 to 400 cm^−1^. Moreover, the data of the powder XRD patterns collected at 2*θ* from 5° to 50° were acquired on a Bruker D8X diffractometer faultlessly fitted out with monochromatized Cu-Kα (*λ* = 0.15418 nm) radiation at room temperature. TGA measurements were carried out on a Diamond thermogravimetric analyzer in a floating N_2_ atmosphere with a measurement interval from 25 to 800 °C accompanied by a heating rate of 10 °C min^−1^.

### Synthesis of [Zn_3_(C_2_H_4_N_4_)_6_(H_2_O)_6_](V_10_O_28_)·14H_2_O (1)

A mixture of V_2_O_5_ (0.1505 g, 0.8 mmol), ZnSO_4_·7H_2_O (0.1498 g, 0.5 mmol), 3-amino-1,2,4-triazole (0.0518 g, 0.6 mmol) and LiOH (0.0603 g, 1.5 mmol) was added to 8 mL of distilled water and stirred for approximately 3 h at room temperature. After stirring well, 2 M acetic acid solution was added to adjust the mixture to about pH = 4. After a few minutes, the insoluble precipitate was removed *via* filtration and the clear solution was evaporated at room temperature. Four days later, orange/yellow block crystals were obtained. Yield: 36% (based on V). Elemental analysis calcd (found%) for the complex: calcd for C, 7.14; H, 3.17; N, 16.65; found for C, 7.24; H, 3.31; N, 16.71.

### Synthesis of [Zn_3_(C_2_H_3_N_3_)_8_(H_2_O)_4_](V_10_O_28_)·8H_2_O (2)

The synthetic procedure for compound 2 was similar to that of 1, except that 1*H*-1,2,4-triazole (0.0520 g, 0.6 mmol) was used to substitute for 3-amino-1,2,4-triazole. Besides this, the evaporation time of complex 2 was much shorter than that of 1. Only one day later, orange/yellow block crystals were obtained. Yield: 44% (based on V). Elemental analysis calcd (found%) for the complex: calcd for C, 9.99; H, 2.50; N, 17.48; found for C, 10.12; H, 2.59; N, 17.53.

### X-ray crystallography

X-ray analysis data for 1 and 2 were obtained on a Bruker Apex II CCD diffractometer at 296 K, with graphite monochromatized Mo-Kα radiation (*λ* = 0.71073 Å). The structures were solved using direct methods and refined *via* full-matrix least-squares using the SHELXL-2014 crystallographic software package. The non-hydrogen atoms were refined anisotropically. During the refinement, the thermal parameters of water and some of the C atoms were restrained. CCDC 1820884 and 1820885.† All the crystallographic information for 1 and 2 is listed in Table 1.

**Table 1:** Crystal data and structure refinements for compounds 1 and 2

Compound	1	2
Formula	C_12_H_64_N_24_O_48_V_10_Zn_3_	C_16_H_48_N_24_O_40_V_10_Zn_3_
Formula weight	2018.38	1922.29
*T* (K)	296(2)	296(2)
Crystal system	Triclinic	Triclinic
Space group	*P*1̄	*P*1̄
*a* (Å)	11.53(3)	10.775(13)
*b* (Å)	11.81(3)	12.464(15)
*c* (Å)	12.99(3)	13.191(16)
*α* (deg)	96.31(3)	117.801(12)
*β* (deg)	110.35(3)	94.002(15)
*γ* (deg)	96.93(4)	103.534(13)
*V* (Å^3^)	1624(8)	1490(3)
*Z*	1	1
*D*_c_ (g m^−3^)	2.064	2.142
*μ* (mm^−1^)	2.574	2.789
*F*(000)	1008	952
*θ* Range (deg)	1.695–25.025	1.870–25.495
Limiting indices	−13 ≤ *h* ≤ 13, −12 ≤ *k* ≤ 13, −15 ≤ *l* ≤ 15	−13 ≤ *h* ≤ 12, −14 ≤ *k* ≤ 15, −15 ≤ *l* ≤ 15
Reflections collected/unique	11 245/5608	10 455/5292
*R* (int)	0.0870	0.0542
Data/restraints/parameters	5553/74/492	5292/48/427
GOF	1.039	1.076
*R*_1_ a, w*R*_2_^b^ [*I* > 2*σ*(*I*)]	0.0773, 0.1561	0.0475, 0.1273
*R*_1_, w*R*_2_ (all data)	0.1542, 0.1780	0.0690, 0.1382

a *R*_1_ = Σ||*F*_o_| − |*F*_c_||/Σ|*F*_o_|.

b w*R*_2_ = Σ[w(*F*_o_^2^ − *F*_c_^2^)^2^]/Σ[w(*F*_o_^2^)^2^]^1/2^.

## Results and discussion

### Synthesis

Over the past few years, the hydrothermal method has been proven to be a cogent approach for the preparation of polyoxovanadates. During a conventional hydrothermal method, numerous factors can impact the nucleation and crystal development of the final products, for example, the initial reactant, the reactant concentration, the solvents, the reaction time, the pH values, and the reaction temperature. However, it is worth noting that we used the volatilization method of adjusting the pH value without heating. The experimental PXRD patterns of the bulk products of compounds 1 and 2 are consistent with the simulated ones calculated from X-ray single-crystal diffraction (Fig. S6 and S7†), which indicates the phase purity of the two compounds. The intensity difference between the experimental and simulated PXRD patterns may be ascribed to the variation in the preferred orientation of the powder sample during measurements. The thermal analysis and IR spectra of both compounds are shown in Fig. S9–S11.†

### Analysis of crystal structures

#### The crystal structure of compounds 1 and 2

**Fig. 1:**
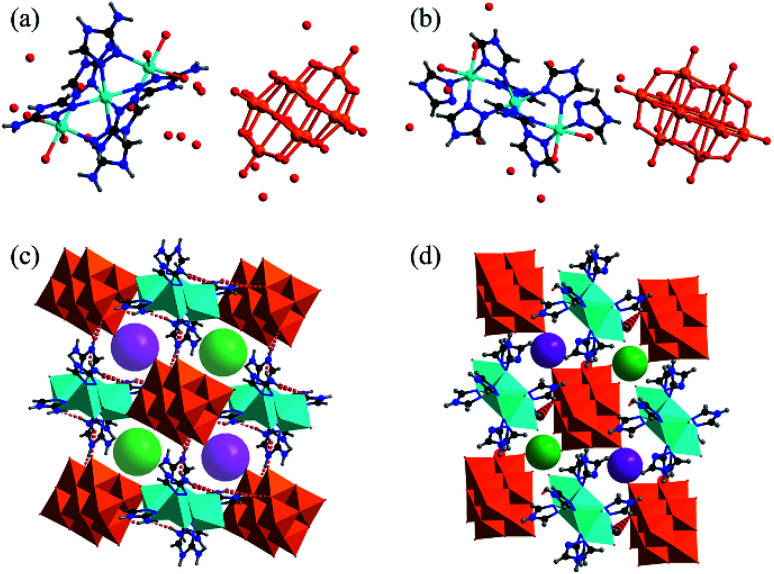
The asymmetric building blocks of complex 1 (a) and complex 2 (b). V: orange; Zn: turquoise; C: black; N: blue; O: red and H: gray. The three-dimensional supramolecular structures of complex 1 (c) and complex 2 (d) connected by hydrogen bonds. The free water molecules were omitted for clarity (violet and green: the sizes of the channels in 1 and 2, found to be 8.58 Å and 3.66 Å, respectively).

Single-crystal X-ray diffraction analysis reveals that 1 and 2 both crystallized in the triclinic space group *P*1̄. The molecular structure of complex 1 consists of isolated [V_10_O_28_] cluster anions, [Zn_3_(C_2_H_4_N_4_)_6_(H_2_O)_6_] coordination cations, and hydration water molecules found among the intervals of the polyanions and cations, as shown in Fig. 1a and b. Compared with 1, the isolated [Zn_3_(C_2_H_3_N_3_)_8_(H_2_O)_4_] cluster cations are found to be dissimilar in complex 2. A typical [V_10_O_28_] cluster consists of ten edge-sharing [VO_6_] octahedra (Fig. S1a†), and the V–O bond distances vary from 1.610(8) to 2.302(8) Å, which are similar to those of the reported decavanadate structure.^11^ There are two crystallographically independent Zn ions (Zn1 and Zn2) in compound 1. Zn2 is six-coordinated by three N atoms from the ligands and three O atoms from the water molecules. Zn1 is located at a symmetry center (1.5, 1.5, 0), bonded by six N atoms from different ligands, and connects two octahedral ZnN_3_O_3_ (Zn2) to form a [Zn_3_(C_2_H_4_N_4_)_6_(H_2_O)_6_] coordination cation as shown in Fig. S1b.† The bond lengths of Zn–N and Zn–O range from 2.086(11) to 2.205(9) Å and from 2.133(9) to 2.207(9) Å, respectively. These are comparable with the reported values of the Zn compounds.^11^

The V_10_O_28_ polyanions and Zn_3_ coordination cations in complexes 1 and 2 are connected to each other by the hydrogen bonds of N–H⋯O (Table S5 and S6†) to form three-dimensional supramolecular structures, as shown in Fig. S3a and S4a.† The N⋯O distances in 1 vary from 2.771(14) to 3.083(14) Å. In the structure of complex 1, each [V_10_O_28_] cluster anion is surrounded by six [Zn_3_(C_2_N_4_H_4_)_6_(H_2_O)_6_]^6+^ cations connected by hydrogen bonds, which generates soft channels (Fig. S3b†).

Nevertheless, because of the smaller steric hindrance of 1*H*-1,2,4-triazole than 3-amino-1,2,4-triazole, in the 3D supramolecular structure of complex 2, each [V_10_O_28_] cluster anion connects eight [Zn_3_(C_2_N_3_H_3_)_8_(H_2_O)_4_] units using hydrogen bonds (Fig. S4b†). Hence, the three-dimensional pores of complex 1 are much larger than those of complex 2. As shown in Fig. 1c and d, the sizes of the channels in 1 and 2 are about 8.6 Å and 3.7 Å, respectively. The above channels in both compounds provide the path for the proton propagation.

#### Proton conductivity

**Fig. 2:**
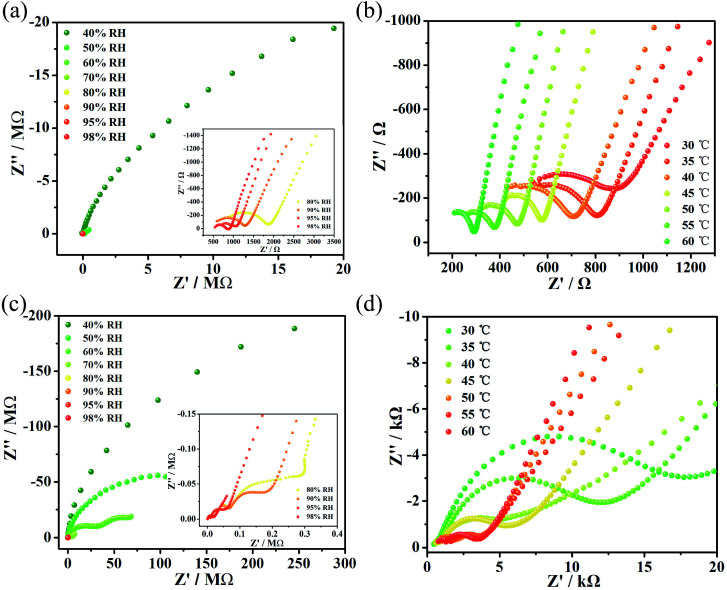
(a) Impedance spectrum of complex 1 under different RH conditions at 30 °C. (b) Impedance spectrum of complex 1 at different temperatures at 98% RH. (c) Impedance spectrum of complex 2 under different RH conditions at 30 °C. (d) Impedance spectrum of complex 2 at different temperatures at 98% RH.

The proton conductivities of complexes 1 and 2 were investigated using alternating current (AC) impedance spectroscopy at diverse temperatures and various levels of humidity. To demonstrate the relation between conductivity and relative humidity (RH), we obtained the conductivities of complexes 1 and 2 at 30 °C under various humidity conditions (Fig. 2a and c). At 40% RH, the conductivity of complex 1 was considered to be an almost inappreciable value of 1.57 × 10^−9^ S cm^−1^. Subsequently, the conductivity value of complex 1 gradually increases to 1.08 × 10^−3^ S cm^−1^ with the increase of humidity to 80% RH. Then, the conductivity value of complex 1 slowly increases with increasing RH in a narrow range of high RH until it reaches 2.67 × 10^−3^ S cm^−1^ at 98% RH. However, the value of proton conductivity for complex 2 is only 9.85 × 10^−5^ S cm^−1^ at 98% RH, which is much lower than that for 1. The increase in proton conductivity with the increase in RH shows that the proton-conducting behavior in the complex is typically controlled by water-mediated proton conduction.^12^

Simultaneously, the proton conductivities of complexes 1 and 2 were also obtained in the temperature range 30–60 °C (Fig. 2b and d) with 5 °C intervals. Finally, the optimal proton conductivities of complexes 1 and 2 were determined to be decent values of 1.24 × 10^−2^ S cm^−1^ and 4.29 × 10^−4^ S cm^−1^, respectively, under 60 °C and 98% RH. What’s more, multiple repetitive tests indicated that the value of the proton conductivity for compounds 1 and 2 could stabilize at 10^−2^ and 10^−4^, respectively. The stability of both compounds after the impedance measurements is shown in Fig. S7 and S8.† For compound 1, we tested the impedance several times and found that its proton conductivity was between 10^−1^ S cm^−1^ and 10^−2^ S cm^−1^, and that the highest value it could reach was 1.25 × 10^−1^ S cm^−1^. However, we chose a more stable value of 1.24 × 10^−2^ S cm^−1^ for reporting. For compound 2, we tested the impedance several times and found that its proton conductivity was between 10^−4^ S cm^−1^ and 10^−5^ S cm^−1^. We chose a more stable value of 4.26 × 10^−4^ S cm^−1^ for reporting.

**Fig. 3:**
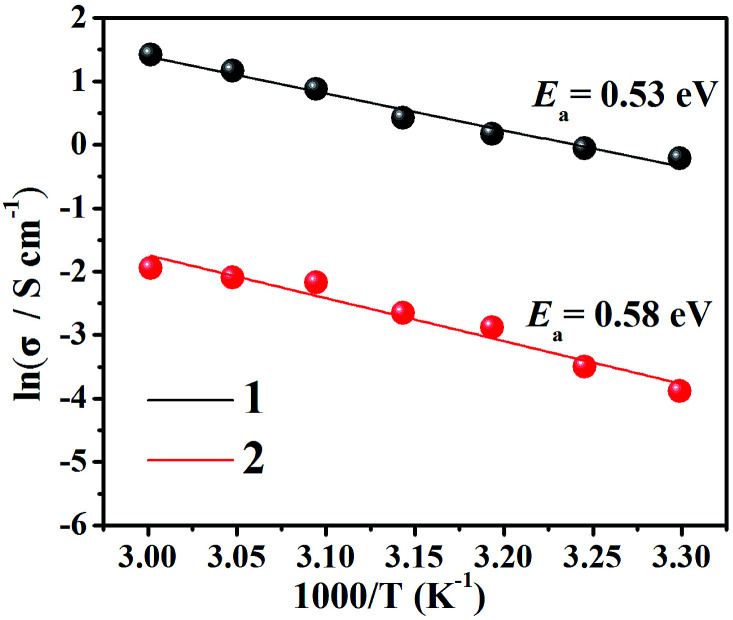
Arrhenius plots of proton conductivity for complexes 1 (black) and 2 (red) under 98% RH conditions.

Moreover, we fitted the slope with least-squares to obtain the activation energy of the complex and to enhance our insights into the proton conduction mechanism (Fig. 3). At 98% relative humidity, the energy of activation (*E*_a_) of complex 1 is 0.53 eV and of complex 2 is 0.58 eV. To explain proton conduction, two cardinal mechanisms (the Grotthuss mechanism and the vehicle mechanism) were used,^13^ as these have a strong association with proton transport. The Grotthuss mechanism establishes the paths for proton conductivity *via* an infinite soft hydrogen bond network and the vehicle mechanism will be considered as only a model that is mainly caused by the movement of protons usually accompanied by the auxiliary movement of a vehicle (*e.g.*, H_2_O, NH_3_). Due to the above characteristics, the two mechanisms can be distinguished *via* the magnitude of the activation energies.^4*a*^ Typically, in the Grotthuss mechanism, the activation energies are in a range of 0.1 to 0.4 eV and correspondingly, the vehicle mechanism can be ascertained from an *E*_a_ of 0.5 to 0.9 eV,^14^ which suggests that the vehicle mechanism may exist in these two complexes.

**Fig. 4:**
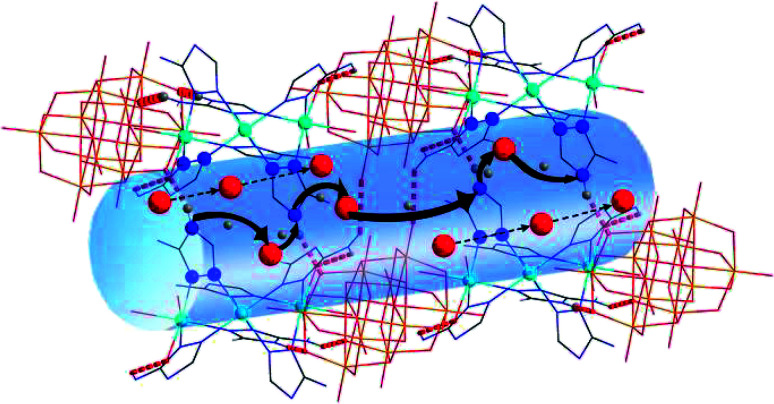
Schematic view of the possible proton-conductive pathways in complex 1. Water molecules are shown in red. The nitrogen atoms of the ligands are shown in blue. The hydrogen atoms of the ligands are shown in gray. The black arrows show the protons hopping along hydrogen bonding networks formed by the coordinated triazole-based ligands and the lattice water. The black dotted arrows represent the transport of protons through the self-diffusion of the protonated water.

According to the structural and activation energy analysis of the two compounds, further insight into the proton channels and mechanisms can be gained from cole–cole fitting (as shown in Fig. S12†). In complex 1, the V–O cluster anions and the [Zn_3_(C_2_N_4_H_4_)_6_(H_2_O)_6_]^6+^ cations were connected by hydrogen bonds to form 3D supramolecular structures (Fig. 4) with narrow channels and a large amount of water moving in a disorderly way in these channels, facilitating proton conduction and thus resulting in a lower activation energy, which is attached to the Grotthuss mechanism. What’s more, six coordinated 3-amino-1,2,4-triazole could contribute abundant protons to the weak N–H bonds, resulting in a high activation energy and a partial vehicle mechanism. That is to say, the proton conductivity of complex 1 combined two mechanisms, the Grotthuss mechanism and a partial vehicle mechanism.^2*b*,15^ Compared to complex 1, although two more coordinated 1*H*-1,2,4-triazoles superseded the two coordinated water molecules in 2, the –NH_2_ of each 3-amino-1,2,4-triazole can provide more plentiful protons. Furthermore, the hydration water molecules in complex 1 are more abundant. At the same time, the size of the pores will directly affect the proton propagation within them, thus further affecting the proton conductivity, and the size of the channels in complex 1 is about 8.6 Å, which is larger than the 3.66 Å channels in complex 2. Hence, the proton conductivity of complex 2 (4.29 × 10^−4^ S cm^−1^) is far less than that of complex 1 (1.24 × 10^−2^ S cm^−1^).

#### Third-order NLO properties

**Fig. 5:**
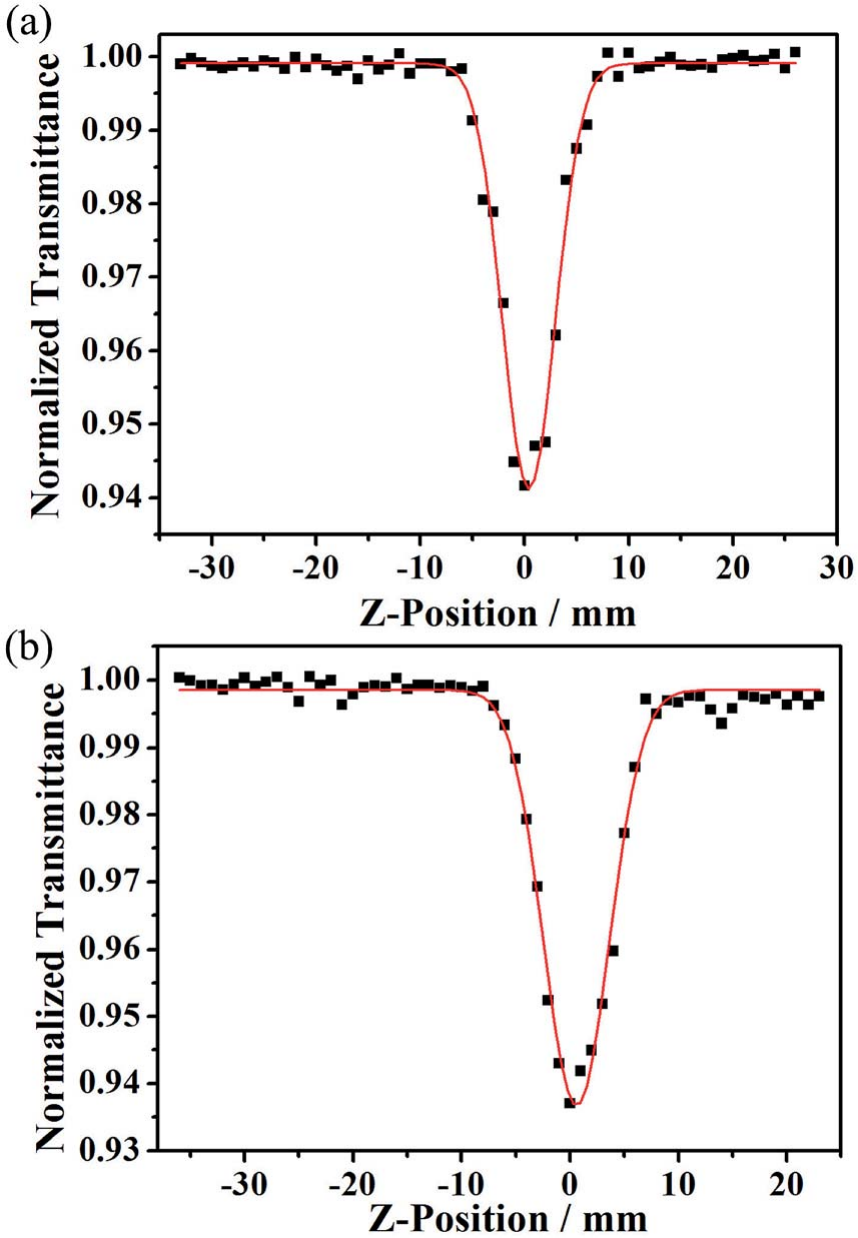
The Z-scan data of 1 in aqueous solution (a) and of 2 in DMSO solution (b), which were acquired under an open-aperture Z-scan. The black squares represent the experimental data, and the red curves were fitted using the theoretical equations.

Since complexes 1 and 2 have triazole-based organic ligands with conjugate units that are decisive for third-order NLO responses, we investigated the solute third-order NLO properties of the two compounds. To measure the third-order NLO responses and two-photon absorption cross sections (*σ*) of compounds 1 and 2, the progressive open-aperture Z-scan technique was adopted. A sample of complex 1 was dissolved in aqueous solution at a concentration of 10–4 mol L^−1^ and measured under a laser beam with a wavelength of 720 nm. However, the sample of complex 2 was dissolved in a solution of DMSO at a concentration of 10–4 mol L^−1^ and measured under a laser beam with a wavelength of 700 nm. As shown in Fig. 5, complexes 1 and 2 were measured using the typical Z-scan method. The experimental data are represented by filled black squares, which can be fitted using the theoretical simulated red light based on the following equations:^16^1
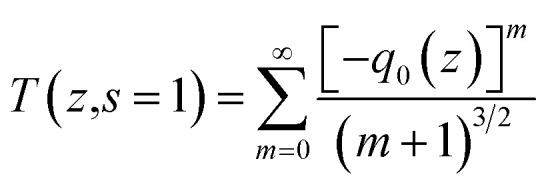
2
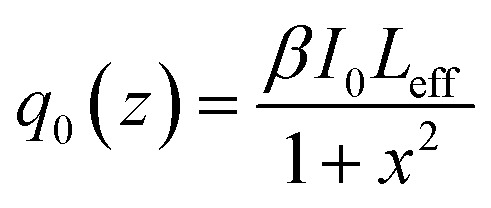
where *I*_0_ is the input intensity, which can be calculated at the focus of *z* = 0 and acquired after dividing the input energy by π*ω*_0_^2^. *L*_eff_ is the effective length, in which *L* represents the sample length and *α* represents the linear absorption coefficient. In the denominator of eqn (2), *x* = *z*/*z*_0_, where *z* stands for the sample position. *z*_0_ = π*ω*_0_^2^/*λ* denotes the diffraction length of the laser beam, in which *ω*_0_ is the spot size at the place of focus, and *λ* is the wavelength of the beam. Besides this, we obtained the NL absorption coefficient *β* using the aforementioned equations. The two-photon absorption cross section *σ* can be calculated using the following evaluation:

3 *σ* = *hνβ*/*N*_A_*d* × 10^−3^

where *hν* is the energy of the incident illumination, *N*_A_ represents Avogadro’s constant, and *d* is the concentration of the complex. By computation, the values of *β* for compounds 1 and 2 are 0.006767 cm GW^−1^ and 0.004494 cm GW^−1^, respectively, and the values of *σ* for compounds 1 and 2 are 3102.13 GM and 2118.82 GM (1 GM = 10^−50^ cm^4^ s per photon), respectively.

## Conclusions

In summary, to investigate the effects of different triazole-based ligands on proton conduction, we successfully prepared two isolated decavanadate-based bifunctional POVs, [Zn_3_(C_2_H_4_N_4_)_6_(H_2_O)_6_](V_10_O_28_)·14H_2_O and [Zn_3_(C_2_H_3_N_3_)_8_(H_2_O)_4_](V_10_O_28_)·8H_2_O. The proton conductivity of complex 1 is much higher than that of complex 2 under the same conditions due to the greater number of lattice water molecules and the protonated –NH_2_ of 3-amino-1,2,4-triazole. The outcome of this work contributes to the understanding of how proton conduction is affected by different triazole-based ligands and will benefit the further investigation of new POV-based proton-conduction materials. Further studies will be focused on the preparation of large single crystals, and the proton conductivity of single crystals with anisotropic characterization along and perpendicular to the channels.

## Conflicts of interest

There are no conflicts to declare.

## Supplementary Material

RA-008-C8RA02694G-s001

RA-008-C8RA02694G-s002
