# Supplementary material for: Proton conductivity resulting from different triazole-based ligands in two new bifunctional decavanadates
Source: RSC Adv. 2018 May 22;8(33):18560–6. doi: 10.1039/c8ra02694g (PMC9080526; doi:10.1039/c8ra02694g)
Supplement: RA-008-C8RA02694G-s001 [file RA-008-C8RA02694G-s001.pdf]

Electronic Supplementary Material (ESI) for RSC Advances.
This journal is © The Royal Society of Chemistry 2018

Supporting Information

Proton-conductivity resulted by Different Triazole-based Ligands in Two New 0D Bifunctional Decavanadate

Jiapeng Cao,‡[a] Fengcui Shen,‡[a,b,c] Ximing Luo,[a] Chenhui Cui,[a] Yaqian Lan*[b] and Yan Xu*[a]

a College of Chemical Engineering, State Key Laboratory of Materials-Oriented Chemical Engineering, Nanjing Tech University, Nanjing 210009, P. R. China. *E-mail: yanxu@njtech.edu.cn

b Jiangsu Collaborative Innovation Centre of Biomedical Functional Materials, Jiangsu Key Laboratory of New Power Batteries, School of Chemistry and Materials Science, Nanjing Normal University, Nanjing 210023, P. R. China.

c College of Biological and Chemical Engineering, Anhui Polytechnic University, Wuhu, 241000, P. R. China.

‡ The authors contributed equally to this work.

Impedance analysis. The samples were put into a homemade mould with a radius of 0.2 cm to get circular pellets. The thickness was measured by a vernier caliper. And then the pellets were smeared by silver colloid on two sides which were fixed on the sample stage with gold wires. The proton conductivities were measured using an impedance/gain-phase analyzer (Solartron S1 1260) over a frequency range from 1 Hz to 1 MHz with an input voltage range from 100 mV to 3000 mV. The measurements were operated at temperatures (25 to 60 °C), with different relative humidities (40 % to 98 % RH). The proton conductivity was calculated using the following equation

$$\sigma = \frac{l}{SR}$$

where σ is the conductivity (S cm^{-1}), l is the thickness (cm) of the pellet, S is the cross-sectional area (cm^2) of the pellet and R is the bulk resistance (Ω). The activation energy (E_a) was calculated from the following equation

$$\ln \sigma_T = \ln \sigma_0 - \frac{E_a}{KT} \qquad (K=8.6\times10^{-5}\ \text{eV/K})$$

where σ is the conductivity (S cm^{-1}), K is the Boltzmann constant (eV/K) and T is the temperature (K).

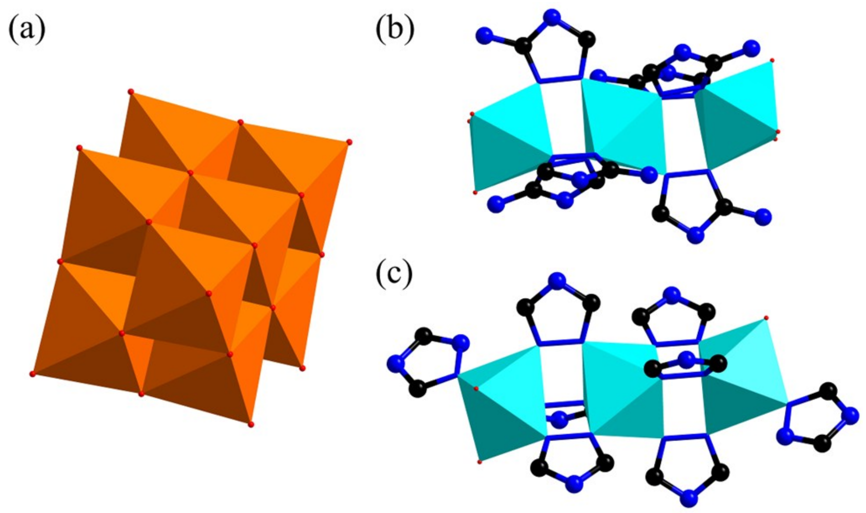

Fig. S1 (a) The $[V_{10}O_{28}]$ cluster with ten V-O octahedron from two complexes, V: orange; O: red. (b) The $[Zn_3(C_2H_4N_4)_6(H_2O)_6]$ cluster from complex **1**, Zn: turquiose; C: black; N: blue; O: red. (c) The $[Zn_3(C_2H_3N_3)_8(H_2O)_4]$ cluster from complex **2**, Zn: turquiose; C: black; N: blue; O: red. (For the sake of clarity, all of the hydrogen atoms and free water molecules have been omitted.)

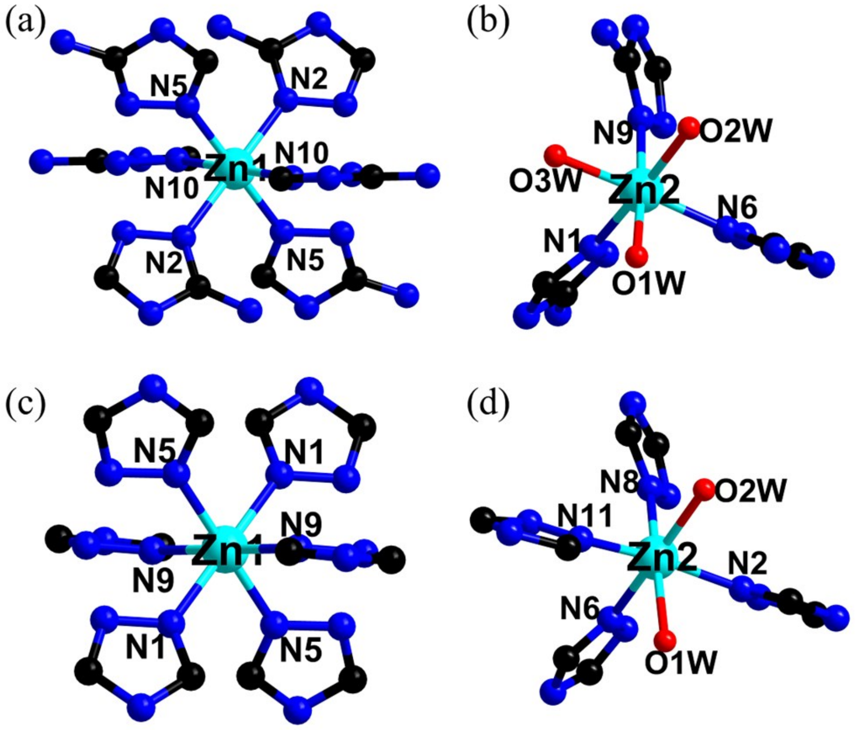

Fig. S2 The coordination mode of Zn1 (a) and Zn2 (b) from the $[Zn_3(C_2H_4N_4)_6(H_2O)_6]$ cluster. And the coordination mode of Zn1 (c) and Zn2 (d) from the $[Zn_3(C_2H_3N_3)_8(H_2O)_4]$ cluster. (For the sake of clarity, all of the hydrogen atoms and free water molecules have been omitted.)

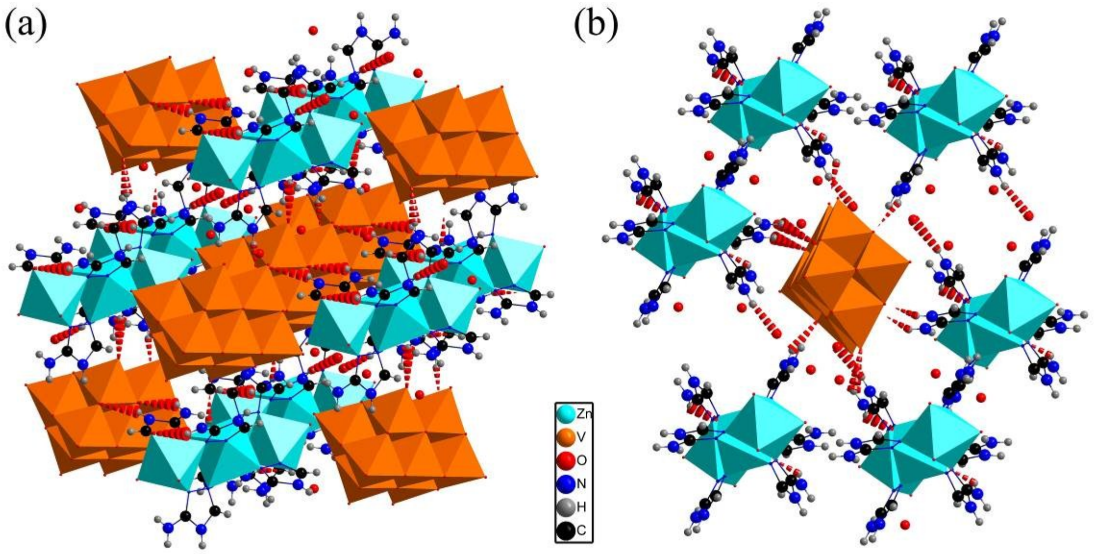

Fig. S3 (a) The 3D supramolecular structure of complex **1** connected by hydrogen bonds. (b) One $[V_{10}O_{28}]$ unit is connected to six $[Zn_3(C_2N_4H_4)_6(H_2O)_6]$ clusters by hydrogen bonds.

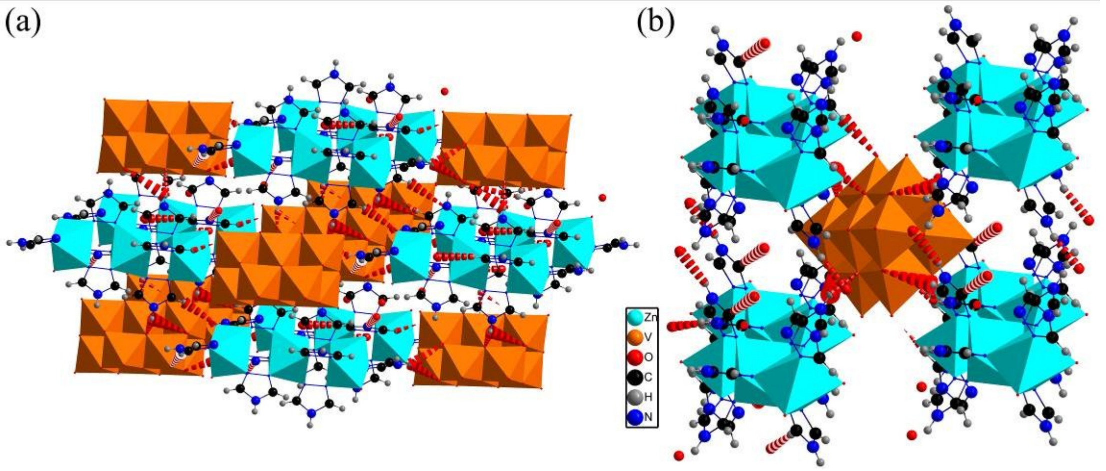

Fig. S4 (a) The 3D supramolecular structure of complex **2** connected by hydrogen bonds. (b) One $[V_{10}O_{28}]$ unit is connected to six $[Zn_3(C_2N_3H_3)_8(H_2O)_4]$ clusters by hydrogen bonds.

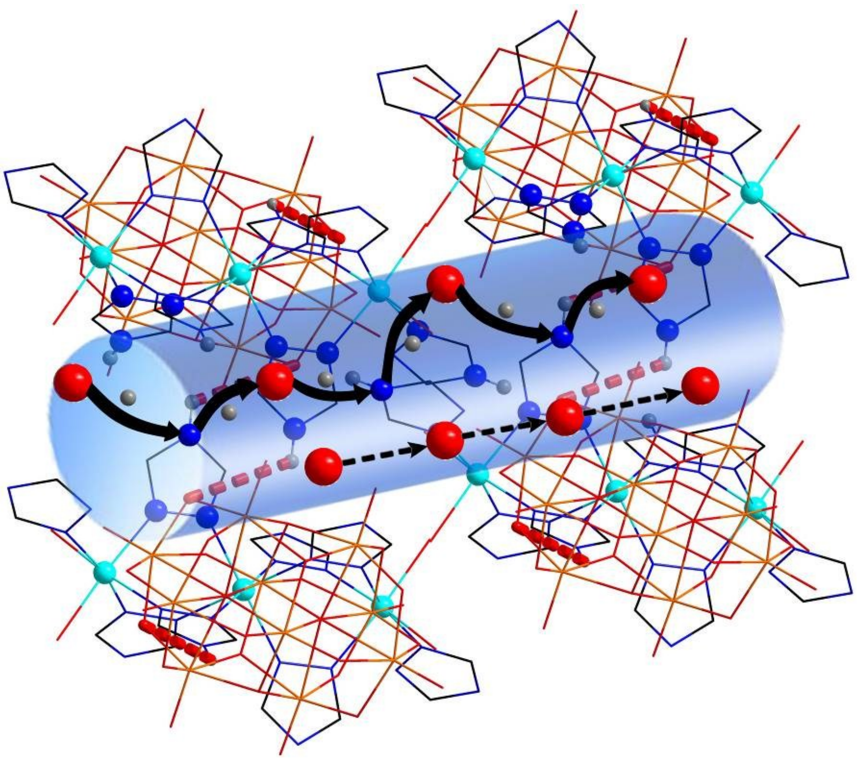

Fig. S5 Schematic view of possible proton-conductive pathways in complex **2**. Water molecules are shown in red. Nitrogen atoms of ligands are shown in blue. Hydrogen atoms of ligands are shown in gray. Black arrows show the protons hop along hydrogen-bonding networks formed by coordinated tridazole-based ligands and lattice water. Black dotted arrows represent transport of protons through self-diffusion of protonated water.

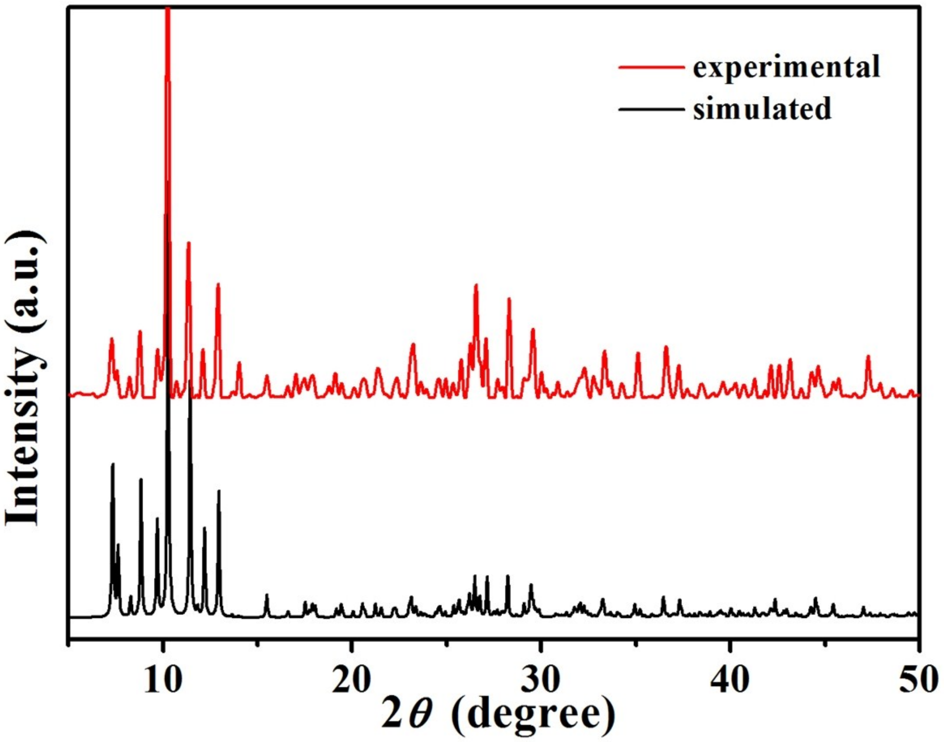

Fig. S6 The powder XRD patterns of complex **1**.

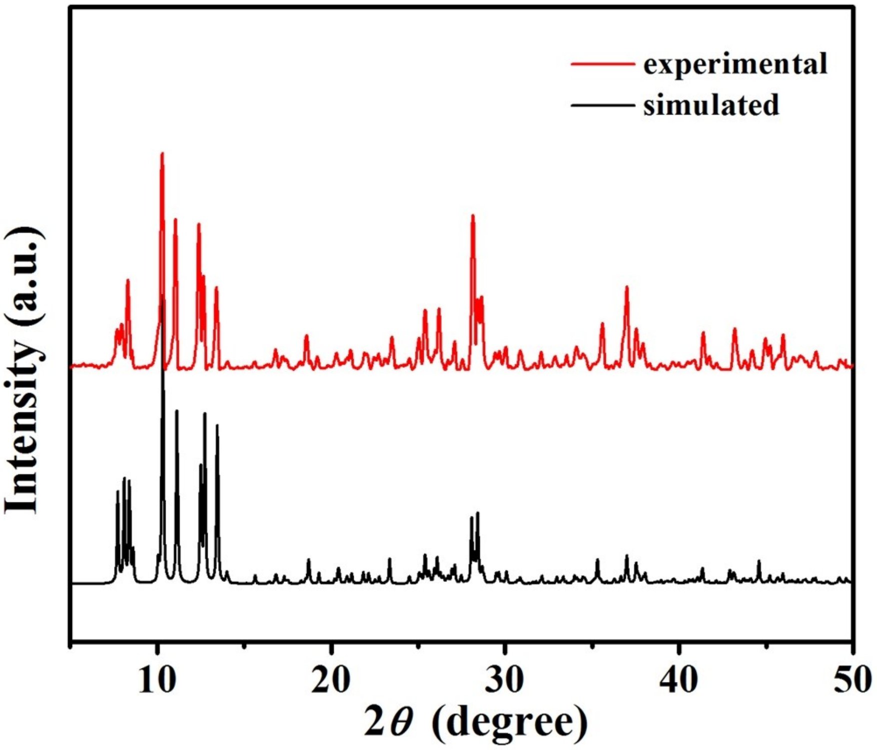

Fig. S7 The powder XRD patterns of complex **2**.

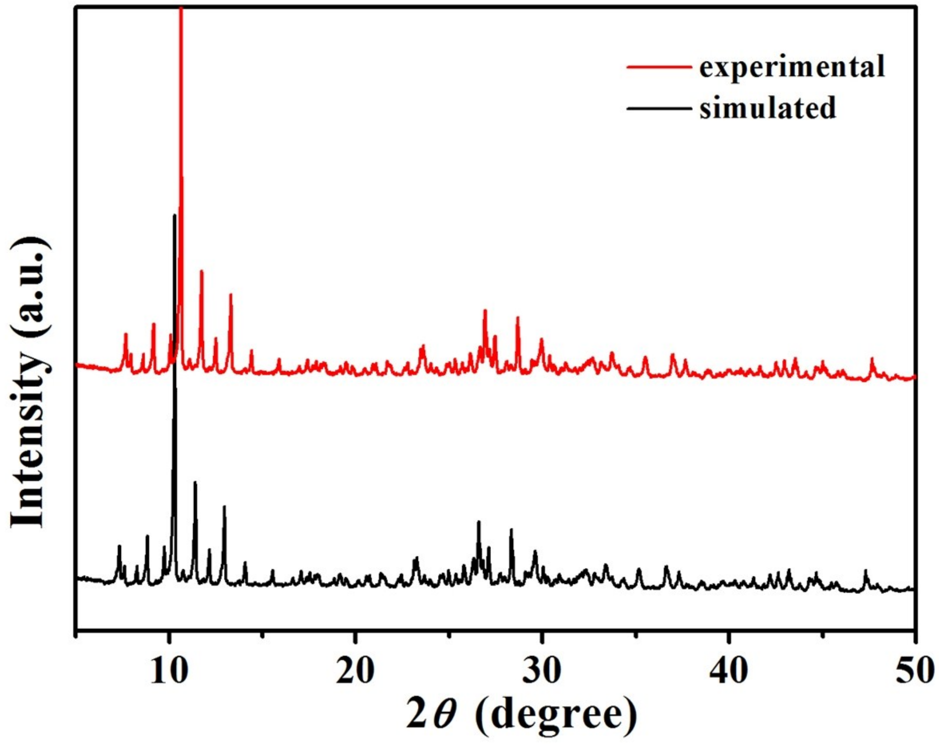

Fig. S7 The powder XRD patterns of complex **1** after impedance measurement.

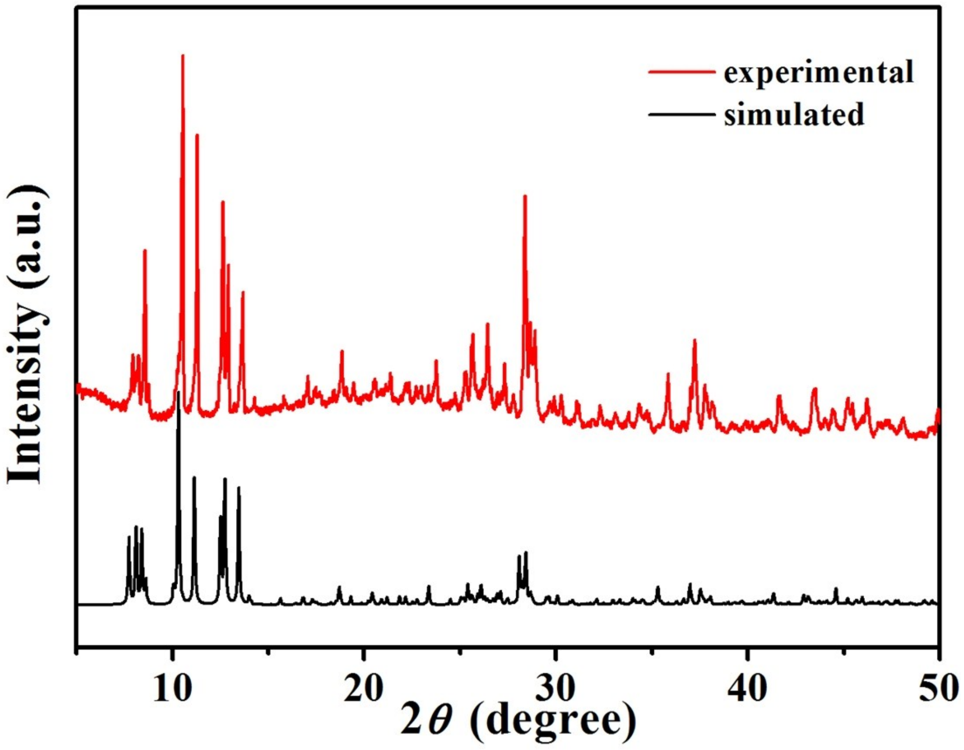

Fig. S8 The powder XRD patterns of complex **2** after impedance measurement.

From the IR spectra, the main peaks of complex **1** and **2** are consistent.

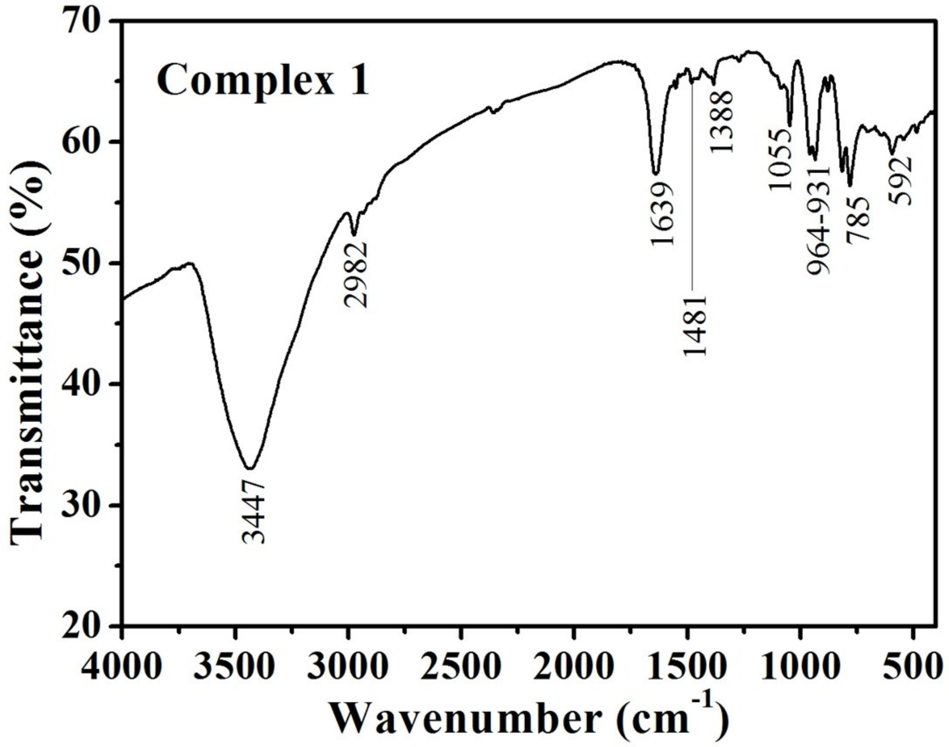

Fig. S9 IR spectra of complex **1**.

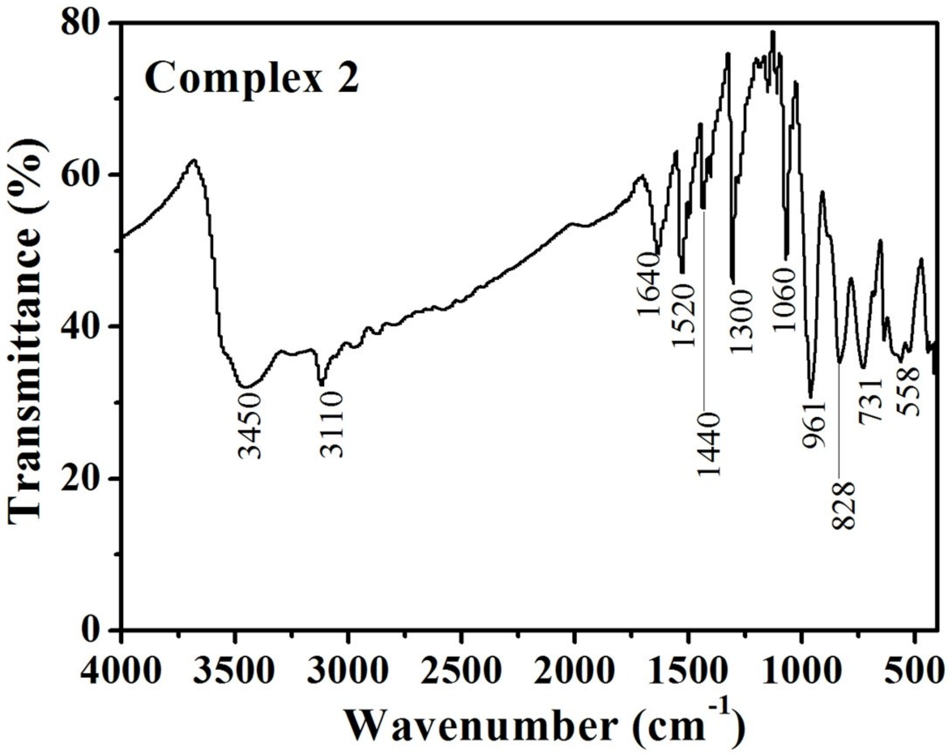

Fig. S10 IR spectra of complex **2**.

In the TG spectra, from 25 to 275 ℃, losing about 22% is ascribed to free water molecules and coordinated water molecules; from 275 ℃ to the end , losing about 28% is ascribed to the decomposition of organic ligands.

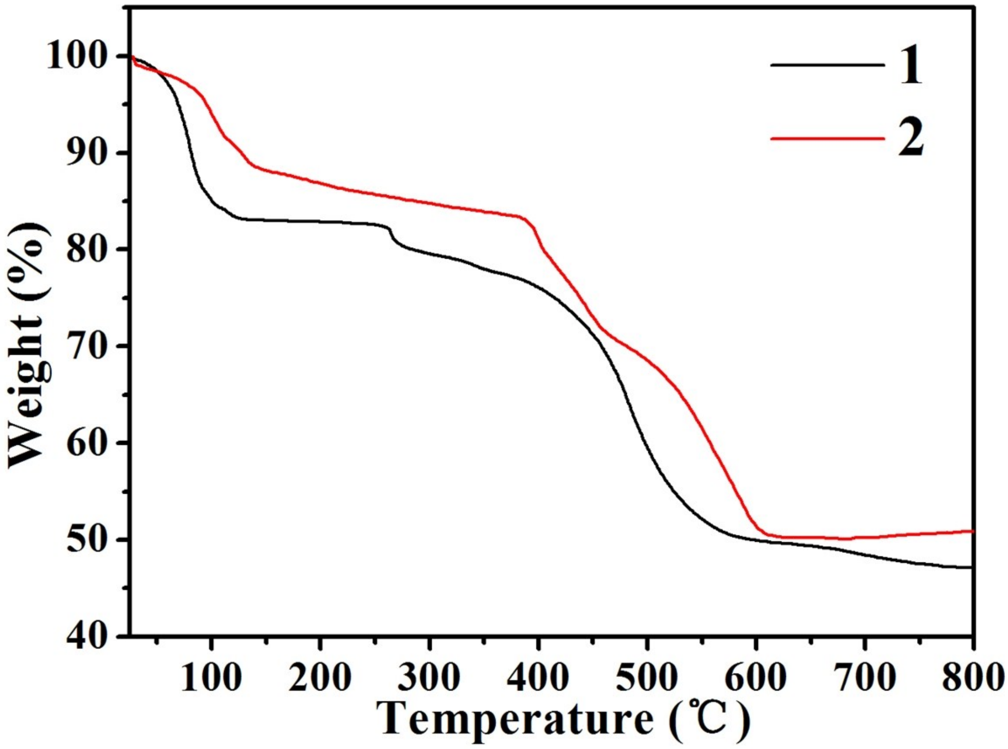

Fig. S11 Thermogravimetric analysis of complexes **1** and **2**.

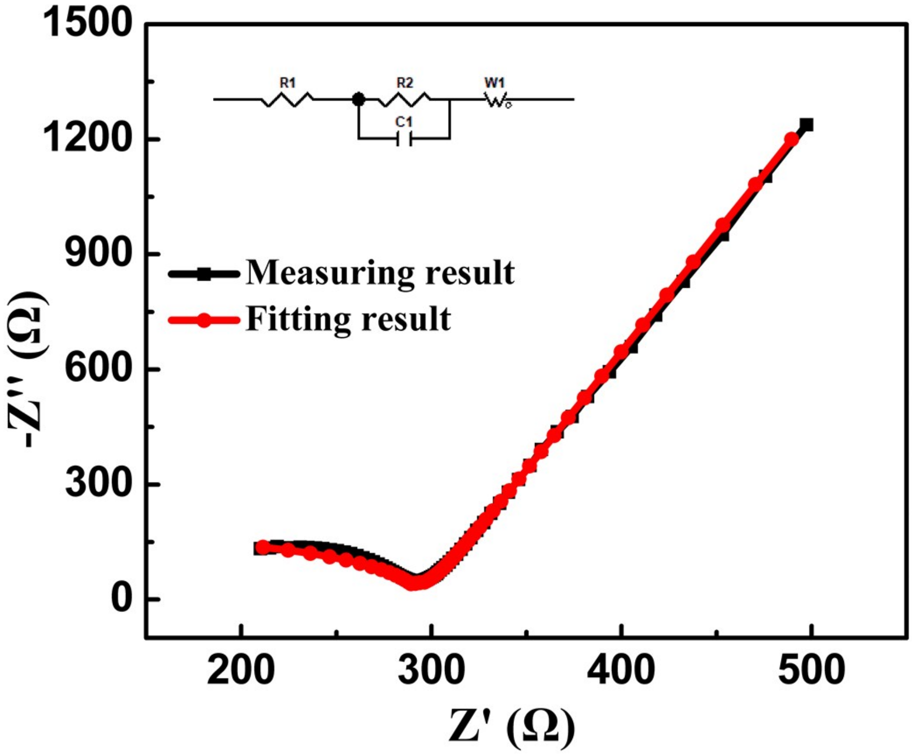

Fig. S12 The cole-cole fitting of proton conductivity.

In Table S1 and S2, the bond lengths around the V atoms of complex **1** are in the range of 1.610(8)-2.302(8) Å (V-O); the bond angles are in the range of 76.0(3)-174.4(3)° (O-V-O). The bond lengths around the Zn atoms are in the range of 2.133(9)-2.207(9) Å (Zn-O) and2.086(11)-2.205(9) Å (Zn-N); the bond angles are in the range of 88.9(3)-180.0° (N-Zn-N), 87.8(3)-172.2(3)° (N-Zn-O) and 86.1(4)-88.4(3)° (O-Zn-O).

Table S1. Bond lengths (Å) of main metal atoms for complex **1**.

Zn(1)-N(10)	2.141(10)	V(4)-O(13)	1.866(8)
Zn(1)-N(2)	2.205(9)	V(4)-O(3)	1.988(8)
Zn(1)-N(5)	2.201(9)	V(4)-O(1)	2.258(8)
Zn(2)-N(9)	2.086(11)	V(5)-O(14)	1.619(8)
Zn(2)-N(6)	2.114(10)	V(5)-O(8)	1.903(8)
Zn(2)-N(1)	2.116(10)	V(5)-O(7)	2.030(8)
Zn(2)-O(1W)	2.133(9)	V(1)-O(11)	1.607(8)
Zn(2)-O(2W)	2.207(9)	V(1)-O(6)	1.842(8)
Zn(2)-O(3W)	2.197(10)	V(1)-O(12)	1.908(8)
V(3)-O(2)	1.690(7)	V(1)-O(2)	2.056(8)
V(3)-O(7)	1.710(8)	V(1)-O(1)	2.302(8)
V(3)-O(5)	1.913(8)	V(2)-O(4)	1.612(8)
V(3)-O(3)	1.941(8)	V(2)-O(10)	1.843(9)
V(3)-O(1)	2.097(8)	V(2)-O(8)	1.830(8)
V(4)-O(9)	1.610(8)	V(2)-O(3)	2.010(8)
V(4)-O(12)	1.809(8)		

Table S2. Bond angles (°) of main metal atoms for complex **1**.

O(2)-V(3)-O(7)	106.2(3)	N(10)-Zn(1)-N(10)#1	180
O(2)-V(3)-O(5)	98.1(4)	N(10)-Zn(1)-N(5)#1	89.4(4)
O(7)-V(3)-O(5)	97.3(3)	N(10)#1-Zn(1)-N(5)#1	90.6(4)
O(2)-V(3)-O(3)	97.2(3)	N(10)-Zn(1)-N(5)	90.6(4)
O(7)-V(3)-O(3)	96.0(3)	N(10)#1-Zn(1)-N(5)	89.4(4)
O(5)-V(3)-O(3)	156.0(3)	N(5)#1-Zn(1)-N(5)	180
O(2)-V(3)-O(1)	88.3(3)	N(10)-Zn(1)-N(2)#1	89.4(3)
O(7)-V(3)-O(1)	165.5(3)	N(10)#1-Zn(1)-N(2)#1	90.6(3)
O(5)-V(3)-O(1)	81.2(3)	N(5)#1-Zn(1)-N(2)#1	91.1(3)
O(3)-V(3)-O(1)	81.0(3)	N(5)-Zn(1)-N(2)#1	88.9(3)
O(9)-V(4)-O(12)	104.0(4)	N(10)-Zn(1)-N(2)	90.6(3)
O(9)-V(4)-O(13)	102.3(4)	N(10)#1-Zn(1)-N(2)	89.4(3)
O(12)-V(4)-O(13)	95.3(3)	N(5)#1-Zn(1)-N(2)	88.9(3)
O(9)-V(4)-O(3)	100.4(3)	N(5)-Zn(1)-N(2)	91.1(3)
O(12)-V(4)-O(3)	90.9(3)	N(2)#1-Zn(1)-N(2)	180
O(13)-V(4)-O(3)	154.2(3)	N(9)-Zn(2)-N(1)	91.8(3)
O(9)-V(4)-O(1)	174.4(3)	N(9)-Zn(2)-N(6)	98.3(3)
O(12)-V(4)-O(1)	80.5(3)	N(1)-Zn(2)-N(6)	92.6(3)
O(13)-V(4)-O(1)	80.3(3)	N(9)-Zn(2)-O(1W)	172.2(3)
O(3)-V(4)-O(1)	76.0(3)	N(1)-Zn(2)-O(1W)	92.8(4)
O(14)-V(5)-O(8)	103.2(4)	N(6)-Zn(2)-O(1W)	87.8(3)
O(14)-V(5)-O(7)	101.1(3)	N(9)-Zn(2)-O(3W)	90.9(3)
O(8)-V(5)-O(7)	83.2(3)	N(1)-Zn(2)-O(3W)	91.9(4)
O(11)-V(1)-O(6)	102.7(4)	O(1W)-Zn(2)-O(2W)	88.4(3)
O(11)-V(1)-O(12)	101.3(4)	O(3W)-Zn(2)-O(2W)	86.1(4)
O(6)-V(1)-O(12)	91.1(3)		
O(11)-V(1)-O(2)	101.0(3)		

In Table S3 and S4, the bond lengths around the V atoms of complex **2** are in the range of 1.603(4)-3.103(4) Å (V-O); the bond angles are in the range of 74.87(14)-173.30(17)° (O-V-O). The bond lengths around the Zn atoms are in the range of 2.184(4)-2.303(6) Å (Zn-O) and 2.059(6)-2.217(5) Å (Zn-N); the bond angles are in the range of 88.45(18)-180.0° (N-Zn-N), 86.74(17)-174.89(15)° (N-Zn-O) and 101.40(19)° (O-Zn-O).

Table S3. Bond lengths (Å) of main metal atoms for complex **2**.

Zn(1)-N(5)	2.133(5)	V(2)-O(11)	1.603(4)
Zn(1)-N(9)	2.193(4)	V(2)-O(10)	1.824(4)
Zn(1)-N(1)	2.217(5)	V(2)-O(8)	1.859(4)
Zn(2)-N(11)	2.059(6)	V(2)-O(2)	2.044(4)
Zn(2)-N(2)	2.130(5)	V(2)-O(1)	2.297(4)
Zn(2)-N(6)	2.135(5)	V(3)-O(12)	1.623(4)
Zn(2)-N(8)	2.138(5)	V(3)-O(6)	1.828(4)
Zn(2)-O(2W)	2.184(4)	V(3)-O(7)	1.829(4)
Zn(2)-O(1W)	2.303(6)	V(3)-O(3)	2.028(4)
V(1)-O(9)	1.696(4)	V(3)-O(1)	2.250(4)
V(1)-O(5)	1.710(4)	V(4)-O(14)	1.612(4)
V(1)-O(2)	1.940(4)	V(4)-O(4)	1.827(4)
V(1)-O(3)	1.961(4)	V(4)-O(9)	2.088(4)
V(1)-O(1)	2.145(3)	V(5)-O(13)	1.612(4)
V(1)-V(4)	3.090(4)	V(5)-O(8)	1.868(4)
V(1)-V(5)	3.103(4)	V(5)-O(6)	1.919(4)

Table S4. Bond angles (°) of main metal atoms for complex **2**.

O(9)-V(1)-O(5)	106.16(19)	N(5)-Zn(1)-N(5)#1	180
O(9)-V(1)-O(2)	98.38(17)	N(5)-Zn(1)-N(9)	91.23(17)
O(5)-V(1)-O(2)	96.13(16)	N(5)#1-Zn(1)-N(9)	88.77(17)
O(9)-V(1)-O(3)	96.94(17)	N(5)-Zn(1)-N(9)#1	88.77(17)
O(5)-V(1)-O(3)	97.30(17)	N(5)#1-Zn(1)-N(9)#1	91.23(17)
O(2)-V(1)-O(3)	155.92(13)	N(9)-Zn(1)-N(9)#1	180
O(9)-V(1)-O(1)	166.57(15)	N(5)-Zn(1)-N(1)	91.55(18)
O(5)-V(1)-O(1)	87.25(18)	N(5)#1-Zn(1)-N(1)	88.45(18)
O(2)-V(1)-O(1)	80.60(14)	N(9)-Zn(1)-N(1)	90.25(17)
O(3)-V(1)-O(1)	80.19(15)	N(9)#1-Zn(1)-N(1)	89.75(17)
O(11)-V(2)-O(10)	104.93(18)	N(5)-Zn(1)-N(1)#1	88.45(18)
O(11)-V(2)-O(8)	102.03(19)	N(5)#1-Zn(1)-N(1)#1	91.54(18)
O(10)-V(2)-O(8)	94.66(19)	N(9)-Zn(1)-N(1)#1	89.75(17)
O(11)-V(2)-O(2)	99.05(19)	N(9)#1-Zn(1)-N(1)#1	90.25(17)
O(10)-V(2)-O(2)	154.30(15)	N(1)-Zn(1)-N(1)#1	180
O(8)-V(2)-O(2)	89.18(17)	N(11)-Zn(2)-N(2)	164.5(2)
O(11)-V(2)-O(1)	173.30(17)	N(11)-Zn(2)-N(6)	93.2(2)
O(10)-V(2)-O(1)	80.66(14)	N(2)-Zn(2)-N(6)	92.05(18)
O(8)-V(2)-O(1)	80.95(15)	N(11)-Zn(2)-N(8)	99.6(2)
O(2)-V(2)-O(1)	74.87(14)	N(2)-Zn(2)-N(8)	94.94(16)
O(12)-V(3)-O(6)	104.84(19)	N(6)-Zn(2)-N(8)	90.87(17)
O(12)-V(3)-O(7)	102.9(2)	N(11)-Zn(2)-O(2W)	89.2(2)
O(6)-V(3)-O(7)	96.24(18)	N(2)-Zn(2)-O(2W)	86.74(17)
O(12)-V(3)-O(3)	99.17(18)	N(6)-Zn(2)-O(2W)	174.89(15)
O(6)-V(3)-O(3)	89.54(17)	O(2W)-Zn(2)-O(1W)	101.40(19)
O(7)-V(3)-O(3)	154.91(16)		
O(12)-V(3)-O(1)	172.88(18)		

Table S5. Hydrogen bonds (Å, °) for complex **1**.

D—H···*A*	*D*—H	H···*A*	*D*···*A*	*D*—H···*A*
O1W—H1WA···O6	0.87	1.98	2.738(14)	145
O1W—H1WB···O8W	0.86	1.84	2.693(15)	171
O2W—H2WA···O11	0.88	2.40	2.864(14)	114
N3—H3C···O5	0.86	2.25	2.989(15)	143
O2W—H2WB···O11	0.88	2.41	2.864(14)	113
N4—H4A···O13	0.86	2.17	2.969(14)	153
N4—H4A···O5	0.86	2.45	3.083(14)	131
O3W—H3WA···O10W	0.85	1.79	2.62(2)	166
O3W—H3WB···O6	0.85	2.08	2.829(14)	146
N7—H7A···O1W	0.86	2.40	3.047(17)	132
N7—H7B···O3	0.86	2.02	2.870(15)	169
N8—H8A···O8	0.86	1.94	2.771(14)	161
O5W—H5WA···O7	0.86	2.39	2.843(14)	113
O5W—H5WB···O7	0.86	2.37	2.843(14)	115
O6W—H6WA···O2W	0.85	2.37	2.734(15)	107
N11—H11A···O4W	0.86	1.96	2.801(14)	167
O6W—H6WB···O2W	0.85	2.44	2.734(15)	101
N12—H12A···O3W	0.86	2.43	3.080(16)	132
N12—H12B···O9	0.86	2.24	2.913(15)	135
O7W—H7WA···O13	0.86	2.29	2.784(14)	117
O7W—H7WB···O8W	0.86	2.50	2.832(17)	104
O8W—H8WB···O5W	0.85	2.21	2.762(16)	122
C5—H5A···O5W	0.93	2.34	3.238(17)	162
C6—H6A···O9W	0.93	2.50	3.40(2)	164

Table S6. Hydrogen bonds (Å, °) for complex **2**.

D—H···*A*	*D*—H	H···*A*	*D*···*A*	*D*—H···*A*
O1W—H1WA···N11	0.87	2.38	2.973(10)	126
O1W—H1WA···O12	0.87	2.46	2.908(9)	113
O1W—H1WB···O12	0.86	2.49	2.908(9)	111
N3—H3B···O5W	0.86	1.96	2.802(7)	166
O2W—H2WB···O6	0.84	2.33	2.685(6)	106
O2W—H2WB···O13	0.84	2.58	3.346(8)	152
N4—H4B···O2	0.86	1.83	2.651(7)	159
O3W—H3WA···O10	0.85	2.29	3.030(8)	146

O3W—H3WB···O1W	0.85	2.57	3.105(11)	122
N7—H7B···O3	0.86	1.91	2.684(7)	149
O5W—H5WA···O7	0.85	1.89	2.734(7)	176
N10—H10A···O4	0.86	2.39	2.886(12)	117
N10—H10A···O8	0.86	2.55	3.300(14)	147
N10—H10A···O10	0.86	2.52	3.282(12)	148
O6W—H6WA···O2W	0.87	2.50	2.990(9)	117
O6W—H6WB···O2W	0.86	2.54	2.990(9)	113
O3W—H3WB···O1W	0.86	2.26	3.066(9)	155
C1—H1A···O4W	0.93	2.48	3.404(12)	171
C3—H3A···O5W	0.93	2.36	3.184(8)	148
C6—H6A···O2W	0.93	2.59	3.506(9)	167
C7—H7A···O12	0.93	2.45	3.351(13)	164
